# Supplementary material for: Association between Weight Status and Mental Health among Korean Adolescents: A Nationwide Cross-Sectional Study
Source: Children (Basel). 2023 Mar 25;10(4):620. doi: 10.3390/children10040620 (PMC10137249; doi:10.3390/children10040620)
Supplement: Supplementary file 1 [file children-10-00620-s001.zip › children-2205857-supplementary.pdf]

Supplementary Table S1. Unadjusted association between characteristics of participants and mental health conditions

	**Depressed mood**				**Perceived stress**				**Suicidal ideation**			
	Male		Female		Male		Female		Male		Female	
	Crude OR (95% CI)	*p* value	Crude OR (95% CI)	*p* value	Crude OR (95% CI)	*p* value	Crude OR (95% CI)	*p* value	Crude OR (95% CI)	*p* value	Crude OR (95% CI)	*p* value
**Age (years)**	1.134 (1.036-1.242)	**0.006**	1.183 (1.094-1.279)	**<0.001**	1.060 (1.005-1.118)	**0.032**	1.124 (1.066-1.184)	**<0.001**	1.067 (0.971-1.173)	0.176	1.067 (0.989-1.150)	0.093
**Total calorie intake (kcal/d)**	1.000 (1.000-1.000)	0.537	1.000 (1.000-1.000)	0.198	1.000 (1.000-1.000)	0.546	1.000 (1.000-1.000)	0.942	1.000 (1.000-1.000)	0.967	1.000 (1.000-1.000)	0.208
**Body mass index**												
Underweight	1.306 (0.755-2.259)	0.340	1.115 (0.643-1.936)	0.698	0.911 (0.629-1.320)	0.623	1.283 (0.867-1.899)	0.213	1.436 (0.726-2.838)	0.298	0.681 (0.324-1.435)	0.313
Normal	1.000		1.000		1.000		1.000		1.000		1.000	
Overweight	1.381 (0.805-2.370)	0.241	1.406 (0.879-2.250)	0.155	1.148 (0.797-1.653)	0.457	1.264 (0.891-1.795)	0.189	1.332 (0.717-2.475)	0.363	1.402 (0.816-2.411)	0.221
Obesity	1.161 (0.644-2.095)	0.619	1.009 (0.618-1.648)	0.971	1.292 (0.950-1.756)	0.102	1.386 (1.009-1.904)	**0.044**	1.345 (0.716-2.527)	0.357	1.080 (0.678-1.719)	0.746
**Intention to control weight**												
No	1.000		1.000		1.000		1.000		1.000		1.000	
Yes	1.179 (0.854-1.629)	0.316	1.563 (1.116-2.190)	**0.009**	1.239 (1.004-1.529)	**0.046**	1.797 (1.426-2.265)	**<0.001**	1.049 (0.684-1.610)	0.826	1.968 (1.313-2.949)	**0.001**
**Perceived health status**												
Very good/ Good	0.946 (0.636-1.406)	0.782	0.679 (0.474-0.974)	**0.035**	0.583 (0.459-0.739)	**<0.001**	0.450 (0.357-0.566)	**<0.001**	0.667 (0.426-1.047)	0.078	0.802 (0.545-1.180)	0.262
Fair	1.000		1.000		1.000		1.000		1.000		1.000	
Very bad/ Bad	3.155 (1.676-5.940)	**<0.001**	3.087 (1.760-5.415)	**<0.001**	2.714 (1.783-4.131)	**<0.001**	2.773 (1.777-4.326)	**<0.001**	4.033 (2.114-7.694)	**<0.001**	4.479 (2.700-7.430)	**<0.001**
**Household income**												
High	0.853 (0.577-1.261)	0.426	0.901 (0.636-1.277)	0.559	0.976 (0.764-1.248)	0.847	1.017 (0.810-1.277)	0.885	1.469 (0.920-2.346)	0.107	0.925 (0.637-1.344)	0.683
Middle	1.000		1.000		1.000		1.000		1.000		1.000	
Low	0.867 (0.484-1.554)	0.632	1.205 (0.761-1.908)	0.427	0.897 (0.606-1.328)	0.588	0.922 (0.665-1.279)	0.628	1.452 (0.681-3.095)	0.334	1.509 (0.951-2.395)	0.081
**Area of residency**												
Urban area	1.000		1.000		1.000		1.000		1.000		1.000	
Rural area	1.309 (0.821-2.086)	0.257	0.855 (0.589-1.242)	0.411	0.869 (0.654-1.156)	0.335	0.851 (0.650-1.115)	0.241	0.886 (0.479-1.640)	0.700	0.756 (0.468-1.221)	0.252

The value was presented as odds ratios (OR) and 95% confidence interval (CI). Significant results are shown in bold.
